# Development of a 3D Co-Culture System as a Cancer Model Using a Self-Assembling Peptide Scaffold

**DOI:** 10.3390/gels4030065

**Published:** 2018-08-02

**Authors:** Nausika Betriu, Carlos E. Semino

**Affiliations:** 1 Tissue Engineering Research Laboratory, Department of Bioengineering, IQS-School of Engineering, Ramon Llull University, 08017 Barcelona, Spain; nausikabetriur@iqs.url.edu; 2 Hebe Biolab S.L. C/Can Castellvi 27, 08017 Barcelona, Spain

**Keywords:** self-assembling peptide scaffold RAD16-I, three-dimensional culture, co-culture system, tumor microenvironment

## Abstract

Cancer research has traditionally relied on two-dimensional (2D) cell culture, focusing mainly on cancer cells and their abnormal genetics. However, over the past decade, tumors have been accepted as complex tissues rather than a homogenous mass of proliferating cells. Consequently, cancer cells’ behavior can only be deciphered considering the contribution of the cells existing in the tumor stroma as well as its complex microenvironment. Since the tumor microenvironment plays a critical role in tumorigenesis, it is widely accepted that culturing cells in three-dimensional (3D) scaffolds, which mimic the extracellular matrix, represents a more realistic scenario. In the present work, an in vitro 3D co-culture system based on the self-assembling peptide scaffold RAD16-I (SAPS RAD16-I) was developed as a cancer model. For that, PANC-1 cells were injected into a RAD16-I peptide scaffold containing fibroblasts, resulting in a 3D system where cancer cells were localized in a defined area within a stromal cells matrix. With this system, we were able to study the effect of three well-known pharmaceutical drugs (Gemcitabine, 5-Fluorouracil (5-FU), and 4-Methylumbelliferone (4-MU)) in a 3D context in terms of cell proliferation and survival. Moreover, we have demonstrated that the anti-cancer effect of the tested compounds can be qualitatively and quantitatively evaluated on the developed 3D co-culture system. Experimental results showed that Gemcitabine and 5-FU prevented PANC-1 cell proliferation but had a high cytotoxic effect on fibroblasts as well. 4-MU had a subtle effect on PANC-1 cells but caused high cell death on fibroblasts.

## 1. Introduction

Classically, cancer research has relied both on two-dimensional (2D) cell culture and on animal models. Animal models are a valuable tool in cancer research. However, they do not represent the behavior of naturally occurring cancers in humans and are expensive, time-consuming, and not feasible for high-throughput screening (HTS) [1,2,3]. On the other hand, 2D in vitro cancer models are under highly controlled conditions and are highly reproducible, which make them very attractive to be used routinely for many research groups and the pharmaceutical industry. Nevertheless, it is widely accepted that 2D models do not represent the in vivo scenario, since cells are grown in a solid and flat support. Thus, cells are forced to polarize, which leads to excessive nutrition and oxygenation, and besides, molecular gradients found in vivo are not reproduced. Moreover, when culturing cells in 2D, the extracellular matrix (ECM)’s composition and configuration are strongly modified, and consequently, cells do not receive the proper signals that provide a normal ECM configuration [4].

Cancer cells, as well as normal cells, require cues from a three-dimensional (3D) environment to form tissue structures in vitro. Moreover, tumor behavior can only be explained considering the contribution of its microenvironment, which includes the ECM (a network of fibrous proteins and proteoglycans), diffusible cytokines and growth factors, the surrounding blood vessels, as well as a set of non-cancer cells, such as fibroblasts. The tumor microenvironment plays an important role in the biology and function of cancer cells, since it provides the surrounding milieu that cancer cells need for survival, growth, proliferation, and metastasis [5,6,7]. Since the microenvironment has a critical role in tumorigenesis, ECM analogs, also called “scaffolds”, have been introduced in 3D cell culture systems. In these systems, cells are embedded in a natural or synthetic scaffold that mimics the ECM, providing thus the chemical, mechanical, and physical cues that cells need to form physiological tissue structures in vitro.

In particular, the self-assembling peptide RAD16-I has been used in the present work as a scaffold for 3D cell culture. RAD16-I is a short peptide constituted by the sequence AcN-(RADA)_4_-CONH_2_, which alternates hydrophilic and hydrophobic amino acids [8,9]. The peptide undergoes self-assembly into a nanofiber network with an antiparallel β-sheet configuration by increasing ionic strength or adjusting pH to neutrality. The nanoscale architecture of the fiber network (around 10 nm diameter and 50–200 nm pore size, a thousand times smaller than mammalian cells) allows the cells to experiment in a truly 3D environment. Besides, biomolecules in such a nanoscale environment diffuse slowly and are likely to establish a local molecular gradient. Non-covalent interactions allow for cell growth, migration, contact with other cells, shape changes, and a proper exposition of membrane receptors [10]. Moreover, since stiffness can be controlled by changing peptide concentration, these hydrogels can be tuned up to embed cells but not to entrap them [9,10]. Since the peptide scaffold does not contain signaling motifs, the environment can be considered non-instructive, from the point of view of cell receptor recognition/activation, making the use of this biomaterial more challenging, since factors that drive tumor progression need to be identified and precisely incorporated. On the other hand, the self-assembling peptide scaffold RAD16-I can be functionalized with signaling motifs, such as ECM ligands for cell receptors, to trigger different cellular responses [11,12].

The aim of the present work is to develop a 3D co-culture system, including not only cancer cells but also stromal cells, to study pancreatic ductal adenocarcinoma (PDAC), which is one of the most aggressive carcinomas [13]. For that, pancreatic ductal adenocarcinoma cells (PANC-1), a well-characterized pancreatic cancer cell line [14], will be co-cultured with human normal dermal fibroblasts (hNDF). With this system, is our intention to recreate a more realistic scenario in which cancer cells would receive from the 3D environment the mechanical and physical signals needed to undergo their biological functions while stromal cells will contribute with the biological signals (such as growth factors and cytokines) that could modulate tumorigenesis. Moreover, since the disadvantage of many anticancer drugs is their non-specificity cell-type activity, co-culture systems are a valuable tool to study the cytotoxic effect of anticancer drugs on normal cells. As proof of principle, the drugs Gemcitabine, 5-Fluorouracil (5-FU), and 4-Methyllumbilliferone (4-MU) will be tested on the developed 3D co-culture system. Gemcitabine and 5-FU block cell-cycle progression at G_1_/S phase, targeting highly proliferating cells [15,16]. On the other hand, 4-MU is a potent inhibitor of hyaluronan synthesis, which has been demonstrated to decrease cell viability, migration, and invasion on different cancer cell types [17,18,19,20,21]. Experimental results showed that Gemcitabine and 5-FU prevented PANC-1 cell proliferation but also had a high cytotoxic effect on fibroblasts. While 4-MU had a subtle effect on PANC-1 cells, it caused high cell death on fibroblasts. With this 3D co-culture system, we were able to evaluate the anticancer effect of these drugs on tumor cells and cytotoxicity on normal cells at the same time, a fact that is valuable given the non-specificity of many anticancer drugs.

## 2. Results and Discussion

### 2.1. Cellular Viability and Proliferation in the 3D SAPS RAD16-I

**Figure 1:**
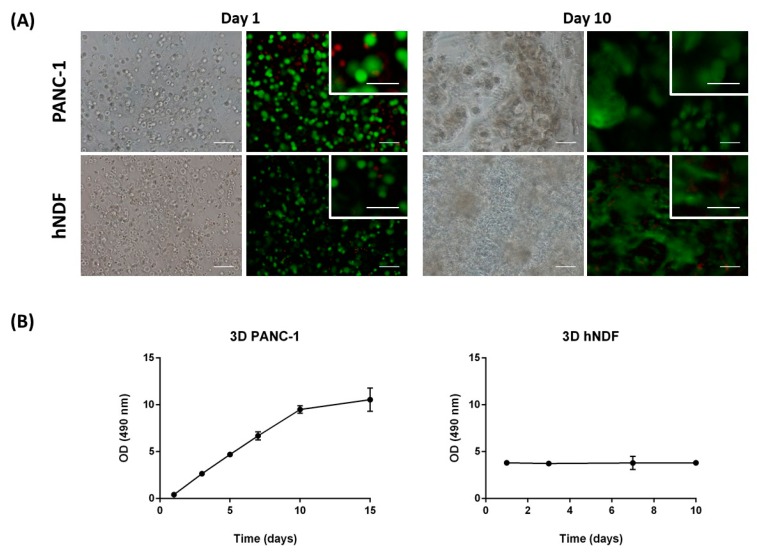
Cellular viability and proliferation in the three-dimensional (3D) SAPS RAD16-I. (**A**) Cellular viability. Viability was assessed at day 0 and 10 of encapsulation into the SAPS RAD16-I. Live cells are stained in green, dead cells in red. Scale bar: 100 µm outside and 50 µm inside the close-up. (**B**) PANC-1 and hNDF growth curves in 3D SAPS. The duplication time obtained for PANC-1 cells was 3 days, while hNDF did not show proliferation. OD, optical density.

PANC-1 cells and hNDF were encapsulated into 3D SAPS and evaluated for viability at day 0 and day 10 of encapsulation. Live/Dead staining shows that few cells died after encapsulation in the SAPS (Figure 1A, left). On the other hand, the death of some cells was probably due to the encapsulation process, where cells experience a short time of low pH of approximately 4–5 (see Section 3). Nevertheless, fluorescence microscopy images of PANC-1 and hNDF cells within the 3D SAPS at day 10 show that most of the cells stay alive along the culture (Figure 1A, right).

PANC-1 and hNDF proliferation within 3D constructs were evaluated through MTS assay. Growth curves for PANC-1 cells in 3D constructs presented approximately a doubling time (Dt) of 3 days (Figure 1B, left). Interestingly, no proliferation was observed for the case of hNDF, suggesting that these cells cultured in 3D configuration enter into G_0_ phase of the cell cycle (Figure 1B, right). Consequently, cellular density increased for PANC-1 cells and it was maintained constant in the case of fibroblasts (Figure 1A). These last ones adopted an elongated shape extending cellular processes within the space of the scaffold, and as a consequence, contracted the entire peptide matrix.

### 2.2. PANC-1 and hNDF 3D Co-Culture Development and Characterization

**Figure 2:**
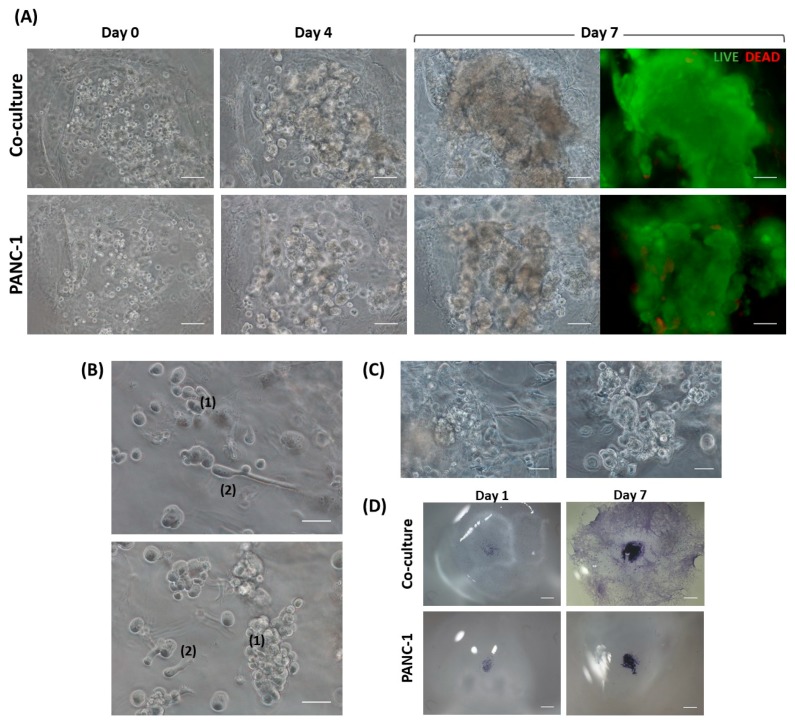
3D culture evolution. (**A**) Tumor core progression in co-culture and PANC-1 monoculture. Comparison of the tumor core in the co-culture (**top**) and PANC-1 monoculture (**bottom**) reveals more compacted PANC-1 clusters when monocultured. Live/Dead staining at day 7 shows high cell viability in both cases. Scale bar: 100 µm. (**B**) PANC-1 phenotypes in 3D co-culture. Close-up sections of the 3D co-culture at day 2 from the injection show epithelial-to-mesenchymal transition in PANC-1 cells. (1) PANC-1 colonies with epithelial phenotype, (2) PANC-1 cells with mesenchymal phenotype. Scale bar: 50 µm. (**C**) Close-up sections of 3D co-culture (**left**) and PANC-1 monoculture (**right**) at day 7. As PANC-1 colonies grow, physical interactions with fibroblasts are established. Scale bar: 50 µm (**D**) Stereoscopic microscopy images of 3D cultures. Co-culture (**top**) and PANC-1 monoculture (**bottom**) stained with toluidine blue at day 1 and 7 from the injection. Scale bar: 1 mm.

Progression of the PANC-1 and hNDF co-culture and the PANC-1 monoculture over 7 days can be easily monitored by visual inspection under microscopy. PANC-1 cell density was significantly increased when co-cultured with hNDF as well as in monoculture. However, PANC-1 cells seemed to form more compacted clusters when monocultured (Figure 2A). This result suggests that PANC-1 tended to migrate more in the presence of fibroblasts. On the other hand, Live/Dead staining at day 7 showed high cell viability in the tumor core in both cases (Figure 2A).

We also analyzed cell phenotype in 3D cultures. Two different PANC-1 cell populations were found: PANC-1 colonies with epithelial phenotype (forming clusters) and single PANC-1 cells with a more mesenchymal phenotype (Figure 2B). This result suggests that PANC-1 cells in this 3D system were able to undergo epithelial-to-mesenchymal transition. On the other hand, interaction between fibroblasts and PANC-1 colonies was established as the tumor grew (Figure 2C, left). Moreover, PANC-1 cells were maintained in 3D along culture time. Fibroblasts, instead, moved from their 3D configuration to the bottom of the gel, highly proliferating in between the cell culture dish and the gel (Figure 2D). This event did not happen in floating gels (Figure 1B). However, the attachment of the gel to the well surface is a requirement for a proper PANC-1 cells injection.

### 2.3. Testing the 3D Co-Culture System with Anticancer Drugs

**Figure 3:**
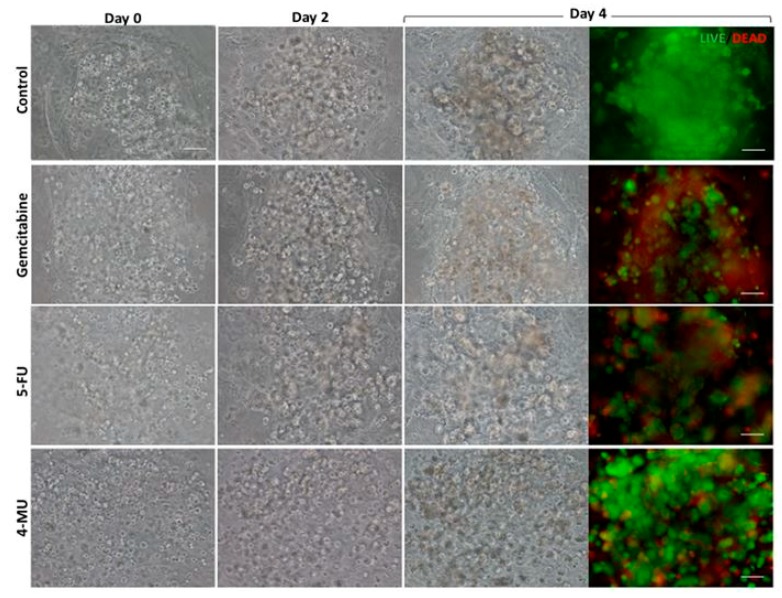
Effect of Gemcitabine, 5-Fluorouracil (5-FU), and 4-Methylumbelliferone (4-MU) on PANC-1 tumor in the 3D co-culture system. PANC-1 cells resuspended in RAD16-I solution were injected into a peptide matrix containing fibroblasts (day 0). Gemcitabine, 5-FU, or 4-MU were added 24 h after the injection. Cells were incubated with the drug for 72 h and assessed for cellular density and viability. Cellular density was evaluated at different time points with phase contrast microscopy, while cellular viability was assessed with Live/Dead at day 4 from the injection. Scale bar: 100 µm.

**Figure 4:**
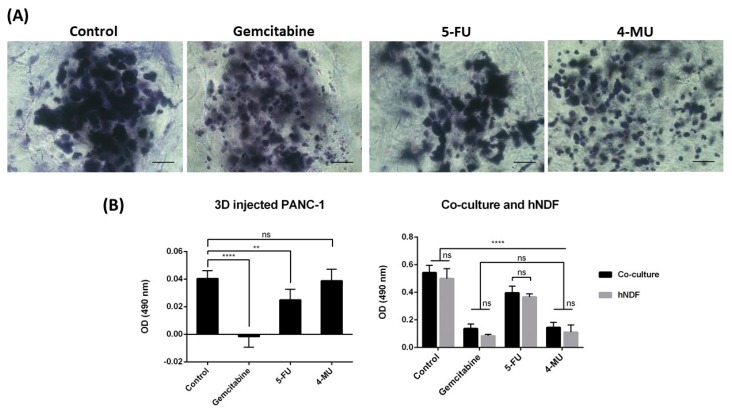
Effect of Gemcitabine, 5-FU, and 4-MU on the 3D co-culture system. (**A**) Toluidine blue staining of 3D co-cultures. Cellular density at day 4 was evaluated using toluidine blue staining. Scale bar: 100 µm. (**B**) MTS assay. MTS was performed on PANC-1 injected in an empty gel (**left**) and on co-cultures and hNDF monocultures (**right**). *N* = 3, *n* = 3.

The effect of Gemcitabine, 5-Fluorouracil (5-FU), and 4-Methylumbelliferone (4-MU) was analyzed in the 3D co-culture system developed. Phase contrast microscopy images of the 3D co-cultures treated with Gemcitabine and 5-FU, which are considered antiproliferative drugs, showed that, as expected, these drugs prevented PANC-1 cell proliferation compared to the control group. Live/Dead staining showed high cell death in the tumor core in both cases, although Gemcitabine seemed to have a more powerful antiproliferative and cytotoxic effect than 5-FU (Figure 3 and Figure 4A). These results were confirmed by MTS assay on injected PANC-1 monocultures (Figure 4B, left). Microscopy images of the 3D co-cultures treated with 4-MU showed less cell density in the tumor core compared to the control (Figure 3 and Figure 4A). However, Live/Dead staining exhibited high cell survival (Figure 3). No differences between the control group and the 4-MU group were observed by MTS assay on injected PANC-1 monocultures (Figure 4B, left). Accordingly, subtle differences regarding cell proliferation cannot be detected by MTS assay but can be perceived by visual inspection.

MTS assay was also performed over 3D co-cultures and hNDF monocultures (Figure 4B, right). Significant differences were found between the control group and all the treated groups in both cases, revealing a high cytotoxic effect of these drugs both over co-cultures and hNDF monocultures. In fact, the optical density (OD) obtained for co-cultures belonged mainly to fibroblasts, since no differences were found between co-cultures and hNDF monocultures OD values. Therefore, the OD produced by PANC-1 cells was not high enough to be detected in the co-culture under these assay conditions. This fact did not surprise us, since the amount of hNDF encapsulated (around 5000 cells) is much greater than the number of PANC-1 cells injected (around 2000 cells). Although it is clear that PANC-1 cells in 3D culture proliferated over time (Figure 2A), the proliferation rate of fibroblast population—that have moved to the bottom and, as a consequence, proliferate in 2D (Figure 2D)—is much higher (~28 h Dt versus ~72 h Dt for PANC-1 in 3D), contributing a majority (around 90%) to the cell mass obtained.

Indirect co-cultures systems, which are based on the use of culture inserts or cell-conditioned medium, are simple and often used for in vitro experiments. However, they are not suitable for investigating the effects of cell contacts between stromal cells and cancer cells. Direct co-cultures models represent more closely the in vivo scenario. However, such systems are more challenging when it comes to quantifying the proliferation and survival of each cell population. Here, we have proposed a 3D co-culture system that allows us to qualitatively assess the effect of drugs on cancer cells (by visual inspection, Live/Dead, and toluidine blue staining) and quantitatively evaluate cytotoxicity on normal cells (by MTS) in the same system. Therefore, this 3D co-culture system is a valuable tool to study the global effect of anti-cancer drugs on tumor and normal cells at the same time. The developed co-culture system could be improved using different cell tracker dyes to label each cell type, and confocal microscopy to obtain a better three-dimensional configuration of cell interactions.

## 3. Materials and Methods

### 3.1. Materials

Human pancreatic ductal adenocarcinoma cells (PANC-1) were purchased from American Type Culture Collection (ATTC, CRL-1469). Primary human normal dermal fibroblasts (hNDF) were purchased from Promocell (C-12302). Cells were cultured in Dulbecco’s modified Eagle’s medium (DMEM, 12491-023; Termo Fisher Scientific, Waltham, MA, USA) supplemented with fetal bovine serum (FBS, S1810; Biowest), penicillin/streptomycin (L0022; Biowest, Nuaillé, France), and L-Glutamine (X0550; Biowest). The 0.05% trypsin-EDTA was from Capricorn Scientific (TRY-1B) and 1× PBS from Biowest (L0615). The self-assembling peptide RAD16-I (1% in water) is available under the commercial name PuraMatrix^TM^ (354250; Corning, New York, NY, USA). Sucrose was purchased from Sigma (S0389, St. Louis, MO, USA). A LIVE/DEAD^®^ Viability/Cytotoxicity Kit for mammalian cells was supplied by TermoFisher Scientific (L3224, Walthan, MA, USA). An MTS Cell Proliferation Assay Kit was purchased from abcam (ab197010). Sigma supplied the drugs Gemcitabine (G6423), 5-Fluorouracil (F6627), and 4-Methylumbelliferone (M1381). Images were taken with a Nikon Eclipse TE2000-1 epifluorescent microscope and a Leica M165C stereoscopic microscope. The statistical software used was GraphPad Prism 6.0 (San Diego, CA, USA).

### 3.2. Methods

#### 3.2.1. 2D Mammalian Cell Culture

Human pancreatic ductal adenocarcinoma (PANC-1) cell line and human Normal Dermal Fibroblast (hNDF) were cultured and expanded in Dulbecco’s modified Eagle’s medium (DMEM) supplemented with 10% fetal bovine serum, 1% penicillin/streptomycin, and 1% l-Glutamine in a humidified atmosphere at 37 °C and 5% CO_2_. Experiments were performed using cell passages between 4 and 20.

#### 3.2.2. Cell Encapsulation into 3D SAPS

For cell encapsulation, we modified a protocol described in the previous work [22]. The commercial peptide RAD16-I (1% in water) was prepared at a final concentration of 0.3% (*w*/*v*) in 10% sucrose and sonicated for 20 min at room temperature. Meanwhile, cells expanded in a 2D monolayer were trypsinized, recollected, and centrifuged (5 min at 60× *g*) and then resuspended in 1–4 mL of 10% sucrose depending on pellet size. Cells were counted and centrifuged again (5 min at 60× *g*). Then, cells were resuspended to 4×10^6^ cells/mL in 10% sucrose. The 0.3% (*w*/*v*) peptide solution was mixed with an equal volume of cell suspension to obtain a final concentration of 2 × 10^6^ cells/mL and 0.15% (*w*/*v*) RAD16-I in 10% (*w*/*v*) sucrose. Next, 40 µL of the mixture (80,000 cells) were loaded into wells of a 48-well plate not treated for cell culture, containing prewarmed media, which induced the spontaneous self-assembling of the peptide. The constructs were left in the flow cabinet for 20 min to let them gel. Then, the media was changed to favor the leaching of sucrose and the equilibration of cells, and the plate containing the 3D constructs was placed in the incubator (37 °C, 5% CO_2_, humidified atmosphere). Cells were maintained in the incubator and the medium was changed every other day.

#### 3.2.3. PANC-1 and hNDF Co-Culture in 3D SAPS

**Figure 5:**
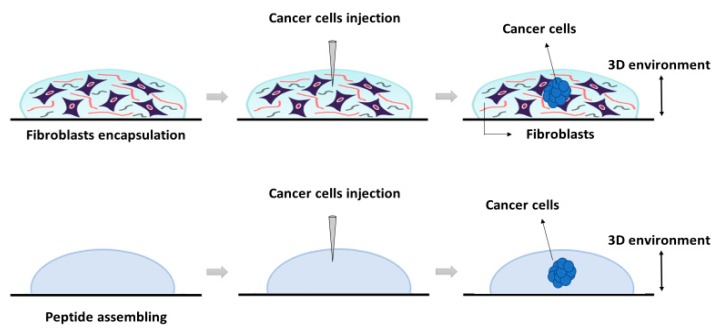
Schematic representation of the 3D system’s construction using the self-assembling peptide scaffold RAD16-I. The system was developed (from left to right) as follows: First, peptide was assembled in presence (**top**) or absence of fibroblasts (**bottom**). Then, cancer cells were injected into each preformed matrix (**center**). The resulting 3D cultures were cancer cells embedded into a fibroblasts matrix (**top**) or a cancer cell into an empty peptide gel (**bottom**).

The construction of the 3D co-culture system consisted of two steps: (1) fibroblasts encapsulation within the RAD16-I peptide scaffold; and (2) cancer cells injection (Figure 5). First, hNDF expanded in a 2D monolayer were harvested by trypsinization, centrifuged (5 min at 60× *g*), and then resuspended in 1–2 mL of 10% sucrose depending on pellet size. hNDF cells were counted and centrifuged again. Then, cells were resuspended in 10% sucrose to 2.5·10^5^ cells/mL. The 0.3% (*w*/*v*) RAD16-I peptide solution (previously sonicated) was mixed with an equal volume of cell suspension to obtain a final concentration of 1.25·10^5^ cells/mL and 0.15% (*w*/*v*) RAD16-I in 10% sucrose. Next, 40 µL of the mixture (5000 cells) were loaded into wells of a 12- or 24-well plate. Medium was added slowly in each well, letting it slide down the wall to the gel, to induce the self-assembling of the peptide. The resulting gel was mechanically attached to the bottom of the well, which both allowed a posterior proper injection and avoided the mechanical contraction produced by fibroblasts growing in 3D gels. The 3D constructs were left in the flow cabinet for 20 min to let them gel. Then, the media was changed to favor the leaching of sucrose and the equilibration of cells, and the plate containing the hNDF 3D constructs was placed in the incubator (37 °C, 5% CO_2_, humidified atmosphere). hNDF were monocultured in the 3D constructs for 3 days.

PANC-1 cells expanded in a 2D monolayer were collected by trypsinization, centrifuged, and then resuspended in 1–2 mL of 10% sucrose depending on pellet size. PANC-1 cells were counted and centrifuged again. Then, cells were resuspended in 10% sucrose to 4·10^6^ cells/mL. The RAD16-I peptide solution (0.3% (*w*/*v*)) was mixed with an equal volume of cell suspension to obtain a final concentration of 2·10^6^ cells/mL and 0.15% (*w*/*v*) RAD16-I in 10% sucrose. Next, 1 µL of the mixture (2000 cells) was injected, using a 10µL micropipette tip, into the 3D constructs containing hNDF cells. PANC-1 cells were also injected and monocultured in an empty gel without fibroblasts. The resulting construction consisted of a rounded gel of about 9 mm diameter, containing or not fibroblasts, with a tumor of approximately 1.5 mm diameter in the center.

#### 3.2.4. Cell Viability

Cell viability was determined using the LIVE/DEAD^®^ Viability/Cytotoxicity Kit for mammalian cells, which presents two components: calcein-AM and ethidium homodimer-1 (EthD-1). The biological principle of this assay is that live cells are distinguished from dead cells by the presence of intracellular esterase activity. The virtually nonfluorescent cell-permeant calcein-AM is cleaved by intracellular esterases, releasing calcein, which is highly fluorescent. The dye calcein is retained in live cells, producing an intense green fluorescence (ex/em 495/515 nm). On the other hand, EthD-1 enters only dead cells, which have damaged membranes. EthD-1 undergoes a 40-fold enhancement of fluorescence when it binds to DNA, producing a red fluorescence in dead cells (ex/em 495/635 nm). Briefly, 3D cultures were rinsed three times with 1× PBS, and then incubated for 40 min in the dark (humidified atmosphere at 37 °C and 5% CO_2_) with a fresh solution of 2 µM calcein and 2 µM ethidium homodimer-1 in PBS. Finally, the samples were rinsed three times with 1× PBS and then visualized under the fluorescence microscope.

#### 3.2.5. Cell Proliferation

An MTS Assay was performed to assess cell proliferation on 3D cell culture. This assay is based on a colorimetric quantification of viable cells. Metabolically active cells reduce the MTS tetrazolium compound to generate a colored formazan product, which can be quantified by measuring the absorbance at 490–500 nm. Briefly, 3D constructs were assembled by triplicate in wells of a 48-well plate. The medium was removed, and 3D constructs were disrupted by pipetting with 1000 µL of a mixture containing MTS reagent and growth medium (20:100). Then, this mixture of cells, medium and reagent, was divided into different wells depending on cell density, and the plate was incubated in the dark for 3 h at 37 °C and 5% CO_2_ in a humidified atmosphere. Next, the content of each well was collected, transferred into separate micro-centrifuge tubes, and centrifuged for 5 min at maximum speed. The aim of this step was to separate the cells and the peptide from the soluble formazan product present in the supernatant. Finally, 100 µL of the supernatant were transferred into a 96-well plate and absorbance was read at 490 nm in a microplate reader. Data was treated as follows: the optical density obtained in each well was subtracted from the blank. Then, the OD values belonging to the same replicate were summed, and finally the mean between replicates and SD were calculated. The doubling time was calculated using Equation (1).

(1) $$Doubling\;time\;(Dt)=\frac{t_{2}-t_{1}}{3.32*(\log D0_{2}-\log D0_{1})}$$

#### 3.2.6. Toluidine Blue Staining

Toluidine blue (TB) is an acidophilic metachromatic dye that stains acidic tissue components (sulfates, carboxylates, and phosphate radicals). Thus, toluidine blue staining can be used as an indicator of proteoglycans (PG) production. However, in the present work, TB staining has been used to evaluate cell density in 3D cultures. For that, 3D constructs were washed with 1× PBS and fixed with 3.7% formaldehyde in PBS for 30 min. Then, cells were washed with 1× PBS and incubated with 0.05% (*w*/*w*) toluidine blue in water for 20 min. Samples were washed several times with distilled water over 24 h and visualized under contrast phase microscopy or a stereoscopic microscope.

#### 3.2.7. Drug Incubation

The chemotherapeutic effect of different drugs was tested on 3D co-cultures, hNDF monocultures, and injected PANC-1 monocultures. The drugs and their concentrations used were: 10 µM Gemcitabine, 10 µM 5-Fluorouracil, and 1 mM 4-Methyllumbilliferone. Drugs were added 24 h after PANC-1 injection into the gel or 4 days after encapsulation in the case of hNDF monocultures. Cells were then incubated with the compounds during 72 h and assessed for viability with a Live/Dead test and an MTS assay.

#### 3.2.8. Statistics

Data are presented as mean ± Standard Deviation. All conditions were tested by triplicate (*n* = 3) in three independent experiments (*N* = 3). One-way ANOVA was used to evaluate statistical differences between experiments. Statistical differences between groups were analyzed using Tukey’s multiple comparisons test at a significance level of 5%. The level of significance was * *p* < 0.05, ** *p* < 0.01, *** *p* < 0.001, **** *p* < 0.0001, ns *p* > 0.05.
